# Supplementary material for: The burden of generational harm due to alcohol use in Tanzania: A mixed method study of pregnant women
Source: PLOS Glob Public Health. 2024 Dec 30;4(12):e0003681. doi: 10.1371/journal.pgph.0003681 (PMC11684658; doi:10.1371/journal.pgph.0003681)
Supplement: Attachment — Submitted filename: review.docx [file pgph.0003681.s003.docx]

# PLOS Global Public Health

## The Burden of Generational Harm due to Alcohol use in Tanzania: a mixed method study of pregnant women

Thank you for letting me review your article, it was an interesting read, and it is a topic worth writing about. I have a large amount of comments, see below. One of the main things I would suggest is to proofread your article again, as there are a multitude of grammatical errors. Furthermore, it is unclear how the research has been conducted. Multiple researches appear to have been conducted simultaneously and then the data has been sifted through to create this article. The sentence “*A thorough overview of the methods has been previously published ^17^.*” made this research all the more confusing. There were also multiple instances of either missing references or incorrect references. I was unable to finish my review due to the large number of issues I found thus far. I have yet to review the tables and the qualitative results.

**Authors**

The order of the authors in the submission box is different from that of the manuscript, be sure to check this before the final submission.

**Keywords**

I would suggest adding a few more, like FASD, etc. This will allow your article will easily be found.

**Abbreviations**

You might want to add FAS in there as well.

**Abstract**

1. The abbreviation ‘*ACDP*’, to denote alcohol consumption during pregnancy, has not been used frequently. Often other authors speak of ‘*prenatal alcohol exposure*’ or ‘*Antenatal alcohol exposure*’. Changing this might increase your findability, and subsequent citation rate.
2. “*Tanzania has minimal policies*…” Please break this sentence up into two separate sentences. This sentence also includes the phrase “*consequent health consequences*”, which is incorrect grammatically. The sentence also speaks of “*pregnancies being exposed to alcohol*”, however it is not the pregnancy being exposed, it is the mother and foetus.
3. “*Research investigating the prevalence of ACDP in SSA —specifically in Tanzania— is abundant.*” Then what is your research setting out to do?
4. “…*alcohol use practices among pregnant women who consume alcohol…*”. You can take out one of the ways to state that they consume alcohol.
5. “…*655 individuals were enrolled*…” were they simply enrolled, or have you recruited them? Did they have a choice?
6. “*…533 female patients presenting for care…*”, please re-word this. You might also want to break up this sentence into two or include a comma.
7. Not sure if you need a comma between sequential exploratory study or a hyphen?
8. Perhaps explain in half a sentence how you went from 655 that have been ‘enrolled’ to having data of 533 patients?
9. You have used the abbreviation IDIs, before explaining what it stands for.
10. “…*were more likely*…” If you explain your results in you abstract and provide percentages, be consistent.
11. “…*highest weekly alcohol expenses*…” How much on average?
12. “*…Average [SD] AUDIT scores …/…Average [SD] PHQ-9 scores…*” If it is correct, your displayed results indicates that there is a large number of outliers, correct?
13. “…Significance…” What you have written here is not the significance of your research, but the conclusion and recommendation. If you are writing about significance, you need to explain what it is that your research uniquely contributes to the understanding of the subject.
14. “…*Our findings demonstrate a concerning trend…”* You cannot speak of a trend without having multiple instances of data collection. ‘Concerning’ indicates a moral judgement, keep it objective where possible.
15. “…*dangers of ACDP are necessary*…” This is statement of fact; however, you are merely recommending this. You can say: “…may proof beneficial”.
16. “…*improve birth experiences*….” With this, are you referring to the use of alcohol during labour? This may just be a lack of accessible adequate healthcare provision. Or is there another reason why alcohol use during pregnancy makes the birth a more negative experience?
17. “…*Further research is needed to explore ACDP as a form of self-medication*…” This has been researched in depth. Is there anything specifically that you think needs to be researched?

**Introduction**

1. “As alcohol use increase globally…” If you look at the statistics globally from the WHO, it shows that alcohol use has gone down every year since 2013. ([Alcohol, total per capita (15+) consumption (in litres of pure alcohol) (SDG Indicator 3.5.2) (who.int)](https://www.who.int/data/gho/data/indicators/indicator-details/GHO/total-(recorded-unrecorded)-alcohol-per-capita-(15-)-consumption))
2. “*Particularly prevalent in low- and middle-income countries, rates of alcohol consumption during pregnancy (ACDP) can range from 2.5% in Nigeria to as high as 32.5% in the Democratic Republic of the Congo (Popova et al., 2016).*” A. you deviated in referencing style here. B. Popova does not give these numbers.
3. “Fetal alcohol spectrum disorder (FASD)” do note that FASD is not an official diagnosis, but an umbrella term of the spectrum of issues children might have developed as a result of prenatal alcohol exposure. In the literature and in the ICD-11 they speak of Fetal Alcohol Syndrome (FAS), which is the official diagnosis. Kruithof, P. and Ban, S., 2021. A brief overview of fetal alcohol syndrome for health professionals. *British Journal of Nursing*, *30*(15), pp.890-893.
4. “…*has an estimated prevalence of 14.8% in Africa, further highlighting alarming rates of ACDP in the region 5,7,8*…” You are referring here to the article by Popova and Lange, the article you have listed as 5. However, if you read the table carefully, it does not say 14.8% of the children have FAS, but that 14.8 in 10.000 children have FAS, so only 0.148%. Also note that they speak of FAS and not FASD.
5. “…*This is increasingly concerning*…” please stay neutral, and you are missing a comma.
6. “*Existing literature emphasizes*…” you then proceed to not reference the literature.
7. “…*the profound health consequences of ACDP in sub-Saharan Africa (SSA)*…” Just in SSA?
8. “…*necessitating increased awareness and robust intervention strategies*…” Is that what the literature suggest or recommends, or is this your own conclusion?
9. Line 108-110 please provide a reference for any claim or statement of fact.
10. “*The lack of intervention and young female education*…” please write this out in full. What kind of intervention? What do you mean with young female education?
11. “…which raises concerns…” please avoid using this type of phrasing, as it is trying to elicit an emotional response. You can use instead ‘may indicate that’.
12. “…, warranting further investigation15…” Is this your conclusion?
13. 115 -120 Please add references.
14. “…*there is a limited understanding of alcohol use practices, the knowledge and attitudes related to ACDP, and the ways these impact cultural beliefs among both pregnant women and women of child-bearing age*…” Some of these things have been researched before, even in the same region. Granted, this is a MSc. thesis, but there are many similarities. Please see the following: Mbuya, B.K., 2022. Factors contributing to drinking alcohol during pregnancy in rural Kilimanjaro Tanzania: a descriptive qualitative study.
15. “This information is needed…” you precede to make a few claims regarding the need for this information, however you have not provided any references.

**Methods**

**Study design overview**

1. “…*focused on exploring unhealthy alcohol consumption at the KCMC ED and RHC*.” Please change: ‘focused on the exploration of alcohol consumption among female patients attending KCMC ED and RHC’. In that way you are not implying people drinking in the hospital. Further, as all alcohol consumption is unhealthy and you are not differentiating between healthy and unhealthy alcohol consumption, it might be best to leave it out.
2. “*The high concentration of injury patients*…” do you mean injured patients?
3. “…*provide ideal settings to study risky alcohol use behaviors*…” Why? The previous claims in the sentence does not explain why this is the ideal setting. It merely explains that there are many coming in with injuries and that there are many women.
4. “*Quantitative surveys and qualitative semi-structured in-depth interviews (IDIs) were conducted, with surveys used to guide purposeful qualitative data sampling.*” Do you mean that the survey have influenced the qualitative interview guide that was used during the IDIs?
5. “*A total of 541 women were enrolled in this study, with 533 providing complete survey data.*” With ‘enrolled’, do you mean ‘approached’, or ‘recruited’ or ‘fell within our criteria and were approached’?
6. “…*totaling 10 female and 9 male IDI participants*…”. Now you stated that this is in an attempt to provide a broader community perspective on the topic, but is this the only reason you interviewed nearly the same amount of men as women? Is the male point of view weighing this heavily in a research which focusses on a female health issue?
7. “*A thorough overview of the methods has been previously published ^17^.*” What do you mean? As these author appear to have done a similar, but definitely different research. Or have you used their research? They also have recruited different number of patients, according to that publication. Were you working side by side? Was there an overlap in your studies? May this have been of influence in the data obtained?

**Exclusion criteria**

1. Why is this a separate heading within the overview, when you are discussing sampling later on?
2. “…*not prisoners*…” Why not? They may have had even more experience with alcohol use. Was there a large female prisoner contingency, which warrant this exclusion criteria?
3. “…*fluent in Kiswahili*,…” Kiswahili or Swahili is the official language beside English, however many other languages are spoken in Tanzania. Were there many that were rejected to participate as a result of this? Did this perhaps alter the sample population, as the other language speaking communities in the region may have given a different point of view within the same area?
4. “*Literature defines the reproductive lifespan of women as 45 years and below^45^.*” I assume this is the wrong reference.

**Quantitative Data**

**Procedure**

1. “*Using intake triage registries from the ED and the RHC,* *a systematic random sampling approach was used to approach every third patient seeking care*…”. I think you probably would have done a pre-selection of the registry, to only include those within the criteria of your research?
2. “*Gender-matched Tanzanian research assistants*…” this is phrase that I have not seen used in academic research before with the meaning that you are intending. Often when gender-matched is used it is referring to data in a cohort. For clarity, as within this chapter, you are solely getting “females” to answer a survey, would continuing the term ‘female’ be better?

**Instruments**

1. “*The Tanzanian research team translated* *reviewed, revised, and pilot-tested all survey questions on sociodemographic data and self-reported alcohol use prior to data collection*.” There are many different steps in translating research tools. How were the translated tools validated?

**Qualitative data**

**Sampling**

1. “…*since data saturation is typically reached around 20 interviews*…” I am unable to find and read the books that you have referenced here. However, it is unlikely that they would give a number.
2. “However, saturation was reached with 19 IDIs, comprising nine males and ten females”
   a. You are missing a period at the end.
   b. You described that there were multiple demographical difference between the interviewees, these being: age, marital status, education level, occupation, tribe, religion, sex, pregnancy status. Of these 8 characteristics, do you say that you have obtained saturation of data until a point where no new data would appear?

**Procedures**

1. “*Upon completion of the quantitative survey, research assistants identified suitable candidates for IDIs using their professional judgment…*”
   a. You stated before that only women took part of the quantitative survey, so how did they identify the men that took part in the qualitative IDIs?
   b. “Using their professional judgement” is a sure fire way to cherry pick data.
2. “…*with monthly reviews of sociodemographic and alcohol-use-related data conducted to ensure diverse representation of participants*.” So did they select based on this data? What did they select for? People who were most represented in the list? What were the points of data in this list?
3. “*Interviewers were fluent in Kiswahili, gender-matched,…*” see previous comments on both topics.

**Instrument**

1. “…*with probes built in for additional questioning when the subject matter was ambiguous, vague, interesting, or inconsistent.*” When you say subject matter, do you mean the interviewee response?
2. “*Cultural appropriateness and relevancy were ensured during pilot testing.*” How? Is this not an exploration of the cultural perceptions in regards to the subject?

**Analysis**

1. “*Qualitative data was analyzed using an applied, thematic, grounded theory approach*” These are different ways of analysing. If you used GT, which version?
2. “*Considering research on pregnant female drinking behavior is limited in Moshi, the grounded theory approach was well-suited to capture emerging themes.*” Please explain what you mean by this.

**Results**

**Quantitative**

1. “*Notable differences in marital status were also observed between pregnant women and non-pregnant women.*” If it is notable, please share what it is.
2. “…*those who were more pregnant were more likely to be living with a partner*…” More pregnant?
3. “…*than older, non-childbearing women.*” So you are pointing out that women aged 17-45 have different characteristics than women aged 45-95. Were you expecting those in the ages above 70 to still be employed?
4. Line 262-264 why are you comparing drinking frequencies between young pregnant women to those not of childbearing age? That is comparing apples with oranges.
5. There are other points in this part where you compare groups who differ on more than one parameter.
6. Line 283 till 285, please move to above the table.
