# Supplementary material for: The burden of generational harm due to alcohol use in Tanzania: A mixed method study of pregnant women
Source: PLOS Glob Public Health. 2024 Dec 30;4(12):e0003681. doi: 10.1371/journal.pgph.0003681 (PMC11684658; doi:10.1371/journal.pgph.0003681)
Supplement: Attachment — Submitted filename: Response to Reviewers.docx [file pgph.0003681.s004.docx]

October 18, 2024

To Whom It May Concern:

On behalf of all authors of this manuscript, we want to express our sincere thanks for the helpful comments and suggestions presented to us by our reviewers. We appreciate the opportunity to improve our manuscript in addressing them and as such, have incorporated all changes as outlined in detail below. Our revised manuscript is attached with this resubmission packet.

Reviewer #1:

I congratulate the authors about this interesting paper. I have only as recommendation to include the entire significance of abbreviations across the paper to facilitate the reading.

*Response: We thank the reviewer for this kind assessment of our manuscript and the very helpful feedback provided. We have fixed all instances where abbreviations were not properly described, to better clarify their significance.*

Reviewer #2:

Thank you for letting me review your article, it was an interesting read, and it is a topic worth writing about. I have a large amount of comments, see below. One of the main things I would suggest is to proofread your article again, as there are a multitude of grammatical errors. Furthermore, it is unclear how the research has been conducted. Multiple researches appear to have been conducted simultaneously and then the data has been sifted through to create this article. The sentence “A thorough overview of the methods has been previously published 17.” made this research all the more confusing. There were also multiple instances of either missing references or incorrect references. I was unable to finish my review due to the large number of issues I found thus far. I have yet to review the tables and the qualitative results.

*Response: We very much appreciate the time and effort you have dedicated to reviewing our manuscript and the helpful suggestions provided below. Per your advice, we have conducted an extensive review of the manuscript, addressing all the concerns you raised. We look forward to your additional comments on the tables and qualitative results.*

**AUTHORS**

**Comment:** The order of the authors in the submission box is different from that of the manuscript, be sure to check this before the final submission.

*Response: We thank the reviewer for bringing this discrepancy to our attention. We have corrected the author list in the submission box to match that of the submitted manuscript.*

**KEYWORDS**

**Comment:** I would suggest adding a few more, like FASD, etc. This will allow your article will easily be found.

*Response: We thank the reviewer for the suggestion to improve the discoverability of our article. We have added the terms "fetal alcohol syndrome" and "fetal alcohol spectrum disorders" to the keywords section. Per your advice below, we have also replaced the keywords "pregnancy" and "alcohol use" with "prenatal alcohol exposure."*

**ABBREVIATIONS**

**Comment:** You might want to add FAS in there as well.

*Response: We appreciate this feedback and have updated the abbreviations section accordingly. Specifically, we have added "FAS: Fetal Alcohol Syndrome" and "FASD: Fetal Alcohol Spectrum Disorders."*

**Comment:** The abbreviation ‘ACDP’, to denote alcohol consumption during pregnancy, has not been used frequently. Often other authors speak of ‘prenatal alcohol exposure’ or ‘Antenatal alcohol exposure’. Changing this might increase your findability, and subsequent citation rate.

*Response: My co-authors and I thank the reviewer for this suggestion. In response, we have replaced all instances of the abbreviation "ACDP" with "prenatal alcohol exposure," "prenatal alcohol use," and/or "alcohol use during pregnancy" throughout the manuscript.*

**ABSTRACT**

**Comment:** (Background) “Tanzania has minimal policies…” Please break this sentence up into two separate sentences. This sentence also includes the phrase “consequent health consequences”, which is incorrect grammatically. The sentence also speaks of “pregnancies being exposed to alcohol”, however it is not the pregnancy being exposed, it is the mother and foetus.

*Response: We appreciate this feedback and acknowledge the grammatical oversight. In line with this feedback, we have modified the sentence, which now reads: "Tanzania has minimal policies, interventions, and educational programs addressing prenatal alcohol exposure. Consequently, a considerable number of mothers and their fetuses are exposed to alcohol, leading to serious health consequences like fetal alcohol spectrum disorder (FASD).”*

**Comment:** (Background) “Research investigating the prevalence of ACDP in SSA —specifically in Tanzania— is abundant.” Then what is your research setting out to do?

*Response: We appreciate the reviewer’s comment and the opportunity to clarify our research focus. As revised in the manuscript, our study “aims to understand the alcohol use practices of pregnant women, the knowledge and attitudes related to prenatal alcohol exposure among different genders and generations, and how these may be influenced by community perceptions and cultural beliefs among patients at Kilimanjaro Christian Medical Center (KCMC).”*

**Comment:** (Background) “…alcohol use practices among pregnant women who consume alcohol…”. You can take out one of the ways to state that they consume alcohol.

*Response: We appreciate the reviewer’s attention to our phrasing and acknowledge this error. We have revised this section to eliminate the redundancy regarding alcohol consumption among pregnant women. This sentence now reads, “Our study aims to understand the alcohol use practices of pregnant women, ...”*

**Comment:** (Methods) “…655 individuals were enrolled…” were they simply enrolled, or have you recruited them? Did they have a choice?

*Response: We thank the reviewer for these thoughtful questions and for highlighting the need for clarification. To clarify, a total of 676 adult patients presenting for care at either the KCMC ED or RHC met our inclusion criteria, were approached for participation using a systematic random sampling method, and were enrolled into the study. While 676 individuals were initially enrolled, only 655 individuals (541 women and 114 men) completed the quantitative survey. That is to say, the individuals who were approached for participation and who were enrolled into the study made an informed decision on whether or not they wanted to participate. Accordingly, this section now reads: “A total of 676 individuals seeking care at the KCMC ED or RHC met our inclusion criteria, were approached for participation using a systematic random sampling method, and were enrolled. Of those, 541 women and 114 men completed the survey.”*

**Comment:** (Methods) “…533 female patients presenting for care…”, please re-word this. You might also want to break up this sentence into two or include a comma.

*Response: We thank the reviewer for this suggestion. We recognize that the sentence was unclear in its original form. As mentioned before, we have edited the methods section of the abstract, addressing this concern. The revised sentence now reads, “Of those, 541 women and 114 men completed the survey. The quantitative analysis focused exclusively on survey data from 533 women who reported both their pregnancy status and age.”*

**Comment:** (Methods) Not sure if you need a comma between sequential exploratory study or a hyphen?

*Response:* *We thank the reviewer for this comment, and we agree that no comma or hyphen is needed between "sequential" and "explanatory" in the description of the study. This has been updated accordingly throughout the manuscript.*

**Comment:** (Methods) Perhaps explain in half a sentence how you went from 655 that have been ‘enrolled’ to having data of 533 patients?

*Response: We thank the reviewer for this suggestion and have made revisions to clarify the recruitment process and how the final dataset of 533 patients for the quantitative analysis was derived from the 655 individuals (541 women and 114 men) who completed the survey. Specifically, the revised manuscript now states, “Of those, 541 women and 114 men completed the survey. The quantitative analysis focused exclusively on survey data from 533 women who reported their pregnancy status and age.” To clarify, for the purposes of this manuscript’s quantitative analysis, all male participants were excluded from the quantitative analysis. We are only using survey data from 533 women who reported both pregnancy status and age, as these two variables are the primary stratification variables for the quantitative analysis to assess alcohol use practices and knowledge among women of different pregnancy statuses and ages.*

**Comment:** (Methods) You have used the abbreviation IDIs, before explaining what it stands for.

*Response: We recognize this oversight and appreciate your comment. We have revised the abstract to include the full term "in-depth interviews (IDIs)" before using the abbreviation.*

**Comment:** (Results) “…were more likely…” If you explain your results in you abstract and provide percentages, be consistent.

*Response: We thank the reviewer for bringing this mistake to our attention. We have revised the results section of the abstract to ensure that percentages are consistently provided wherever necessary.*

**Comment:** (Results) “…highest weekly alcohol expenses…” How much on average?

*Response: We thank the reviewer for this question. Our results section reports weekly alcohol expenses using a categorical variable, so we have included the relevant information in the revised sentence. The updated sentence now reads: “This group [younger non-pregnant women] also reported the highest weekly alcohol expenses, with 18.1% spending over 10,000 Tanzanian shillings (TZS) per week.”*

**Comment:** (Results) “…Average [SD] AUDIT scores …/…Average [SD] PHQ-9 scores…” If it is correct, your displayed results indicates that there is a large number of outliers, correct?

*Response: We thank the reviewer for bringing this to our attention. After careful review, we recognize that our AUDIT, DrInC and PHQ-9 score data does contain several outliers. We have also evaluated the distribution of this data, whereby these variables appear to be non-normally distributed. Therefore, to provide a more accurate representation of the central tendency and variability, we have replaced the mean [SD] for these variables with the median [interquartile range (IQR)] for the AUDIT, DrInC, and PHQ-9 scores in the abstract, results, and discussion sections of the manuscript. Additionally, we have used the Kruskal-Wallis tests and Dunn's test for post hoc pairwise comparisons to compare the medians among pregnant women, younger non-pregnant women, and older non-pregnant women, rather than using the Analysis of Variance (ANOVA) test.*

**Comment:** (Significance) “…Significance…” What you have written here is not the significance of your research, but the conclusion and recommendation. If you are writing about significance, you need to explain what it is that your research uniquely contributes to the understanding of the subject.

*Response: We appreciate this comment from the reviewer and agree that the text provided in the "Significance" section was actually more focused on conclusions and recommendations, which was our intention. However, we mistakenly labeled this section as "Significance." To correct this, we have relabeled the section to "Conclusions" to accurately reflect its content.*

**Comment:** “…Our findings demonstrate a concerning trend…” You cannot speak of a trend without having multiple instances of data collection. ‘Concerning’ indicates a moral judgement, keep it objective where possible.

*Response: We thank the reviewer for this feedback and will be more careful to maintain objectivity and accuracy in our writing. We have revised the sentence to objectively present our findings. The revised sentence now reads, “Our findings demonstrate high rates of prenatal alcohol use in Moshi, Tanzania, with pre-pregnancy alcohol use as a significant predictor.”*

**Comment:** “…dangers of ACDP are necessary…” This is statement of fact; however, you are merely recommending this. You can say: “…may proof beneficial”.

*Response: We thank the reviewer for this suggestion and have revised the sentence accordingly. The revised sentence now reads: "To improve health outcomes and reduce alcohol-related pregnancy complications for current and future generations, community-wide health messaging and pre-pregnancy interventions may prove beneficial for pregnant women and women of reproductive age who consume alcohol."*

**Comment:** “…improve birth experiences….” With this, are you referring to the use of alcohol during labour? This may just be a lack of accessible adequate healthcare provision. Or is there another reason why alcohol use during pregnancy makes the birth a more negative experience?

*Response: We appreciate the reviewer's insightful comment. To clarify, we were not referring to the use of alcohol during labor. We were suggesting that women in Moshi, Tanzania, may resort to alcohol use before and during labor due to its perceived benefits in reducing pain during vaginal birth and alleviating fear and anxiety leading up to childbirth, especially in the context of limited healthcare services and medications for antenatal care and labor. Our intent was to suggest that accessible and adequate healthcare services for pregnant women, such as alcohol-risk screening, alcohol reduction counseling, education on the harms of prenatal alcohol exposure, and readily available medications for pain management during vaginal births, could reduce the reliance on alcohol as a method of managing anxiety, fear, and pain associated with the birth experience. However, we have revised the conclusion of the abstract to exclude this information and focus more directly on the core findings of our study as it relates to our recommendations. This section now states, “To improve health outcomes and reduce alcohol-related pregnancy complications for current and future generations, community-wide health messaging and pre-pregnancy interventions may prove beneficial for young women and pregnant women who consume alcohol.”*

**Comment:** “…Further research is needed to explore ACDP as a form of self-medication…” This has been researched in depth. Is there anything specifically that you think needs to be researched?

*Response: We appreciate the reviewer's comment and acknowledge that the use of alcohol as a form of self-medication has been researched in depth. To answer your thoughtful question, we believe there is a need for more focused research on the specific use of alcohol among pregnant women in SSA for stress and addiction management. As previously stated, we have revised the conclusion of the abstract to exclude this information and focus more directly on the core findings of our study, and related recommendations regarding community-wide health messaging and pre-pregnancy intervention development. Nonetheless, this information is discussed in our discussion.*

**INTRODUCTION**

**Comment:** “As alcohol use increase globally…” If you look at the statistics globally from the WHO, it shows that alcohol use has gone down every year since 2013. (Alcohol, total per capita (15+) consumption (in litres of pure alcohol) (SDG Indicator 3.5.2) (who.int))

*Response: We thank the reviewer for their comment and cross-checking this reference. We acknowledge the recent WHO statistics which indicate that alcohol use has gone down every year since 2013. However, there remains existing literature that supports the claim that alcohol use is increasing globally. Specifically, Manthey et al. (2019) in The Lancet reported that between 1990 and 2017, global adult per-capita consumption increased from 5.9 L to 6.5 L and is forecasted to reach 7.6 L by 2030 — a 70% increase. This trend is driven largely by increases in alcohol consumption in low- and middle-income countries. Thus, while global per capita consumption may have seen a recent decline, the broader trend over the past few decades and in specific regions underscores an overall increase in alcohol use. This is further supported by one of our original references (e.g., Shield et al. (2020)). We have updated our manuscript to include these references to better support our original sentence.*

**Comment:** “Particularly prevalent in low- and middle-income countries, rates of alcohol consumption during pregnancy (ACDP) can range from 2.5% in Nigeria to as high as 32.5% in the Democratic Republic of the Congo (Popova et al., 2016).” A. you deviated in referencing style here. B. Popova does not give these numbers.

*Response: We thank the reviewer for this comment and recognize this mistake in our referencing style. We have made the necessary corrections. Regarding the statistics, the manuscript titled “Actual and predicted prevalence of alcohol consumption during pregnancy in the WHO African Region” by Popova et al. does provide these figures. This article specifically states, “The prevalence of alcohol consumption during pregnancy among the general population in the 25 identified studies ranged from 2.5% (Nigeria) to 32.5% (Democratic Republic of the Congo).” Nonetheless, we have conducted a comprehensive review of the entire manuscript for any incorrect or missing references and have made all necessary adjustments to ensure accuracy, including in this instance.*

**Comment:** “Fetal alcohol spectrum disorder (FASD)” do note that FASD is not an official diagnosis, but an umbrella term of the spectrum of issues children might have developed as a result of prenatal alcohol exposure. In the literature and in the ICD-11 they speak of Fetal Alcohol Syndrome (FAS), which is the official diagnosis. Kruithof, P. and Ban, S., 2021. A brief overview of fetal alcohol syndrome for health professionals. British Journal of Nursing, 30(15), pp.890-893.

*Response: We thank the reviewer for this insightful comment and for sharing this reference. We have made sure to emphasize this concept through the remainder of our manuscript.*

**Comment:** “…has an estimated prevalence of 14.8% in Africa, further highlighting alarming rates of ACDP in the region 5,7,8…” You are referring here to the article by Popova and Lange, the article you have listed as 5. However, if you read the table carefully, it does not say 14.8% of the children have FAS, but that 14.8 in 10.000 children have FAS, so only 0.148%. Also note that they speak of FAS and not FASD.

*Response: We thank the reviewer for bringing this to our attention and acknowledge the error in our manuscript. Upon re-evaluation, we agree that the correct figure reported by Popova and Lange is indeed 0.148% (14.8 per 10,000 children) for FAS, not 14.8%. To more accurately represent the rate of FASD in the region, we have updated the reference used. Specifically, May et al. report “... FASD prevalence remains high at 16% to 31%” in South Africa. Accordingly, our manuscript now reads, “FASD is often underdiagnosed in Africa, with prevalence rates in South Africa ranging from 16 – 31%.” This adjustment ensures our manuscript accurately reflects the existing literature on FASD prevalence in a region of Africa.*

**Comment:** “…This is increasingly concerning…” please stay neutral, and you are missing a comma.

*Response: We appreciate the reviewer’s helpful feedback and recognize the oversight. We have revised the sentence to maintain a neutral tone and correct the grammar mistake. The revised sentence now reads: “Populations with high rates of binge drinking have been linked to similarly high rates of Fetal Alcohol Spectrum Disorder (FASD).”*

**Comment:** “Existing literature emphasizes…” you then proceed to not reference the literature.

*Response: We appreciate the reviewer's observation. The statement was intended to reference the literature discussed in the first two paragraphs of the introduction, as well as additional sources. To address this, we have added specific citations to the sentence.*

**Comment:** “…the profound health consequences of ACDP in sub-Saharan Africa (SSA)…” Just in SSA?

*Response: We appreciate the reviewer’s question and recognize that alcohol consumption during pregnancy is a global issue. We initially focused on sub-Saharan Africa (SSA) in this sentence to emphasize the specific context of Tanzania, which is the focus of our paper. However, we have revised the sentence to highlight that prenatal alcohol use is a public health concern worldwide: “Existing literature emphasizes the profound health consequences of prenatal alcohol exposure globally and in sub-Saharan Africa (SSA), necessitating increased awareness and robust intervention strategies.”*

**Comment:** “…necessitating increased awareness and robust intervention strategies…” Is that what the literature suggest or recommends, or is this your own conclusion?

*Response: We thank the reviewer for this question. The need for “increased awareness and robust intervention strategies” is a statement recommended by the existing literature, and we acknowledge that we did not originally include references for this. We have revised the sentence to include appropriate citations.*

**Comment:** Line 108-110 please provide a reference for any claim or statement of fact.

*Response: We thank the reviewer for their careful examination of our references. We recognize oversight in not including citations for these claims. We have conducted a comprehensive review of the manuscript and have now added the necessary citations to ensure the accuracy of all statements made.*

**Comment:** “The lack of intervention and young female education…” please write this out in full. What kind of intervention? What do you mean with young female education?

*Response: We thank the reviewer for these questions and the opportunity to clarify. We have revised the sentence to specify the types of interventions and the nature of young female education we were originally referring to. Evidence has indicated that regular and/or harmful alcohol use behaviors among women of reproductive age in sub-Saharan Africa (SSA) often continue into pregnancy. These harmful behaviors are further encouraged by minimal policies and limited knowledge about the dangers of alcohol use during pregnancy.*

*Therefore, we were specifically referring to the lack of ‘targeted alcohol-reduction’ interventions for young women of childbearing age and for pregnant women, as well as insufficient community-based education on prenatal alcohol exposure, both of which contribute to the continued consumption of alcohol by pregnant women in Tanzania.*

*The revised sentence makes this clearer and now reads: “The lack of public health interventions aimed at pregnant women and women of childbearing age —including alcohol prevention programs and community-based education on prenatal alcohol exposure— contributes to a significant number of mothers and their fetuses in Tanzania being exposed to alcohol.”*

**Comment:** “…which raises concerns…” please avoid using this type of phrasing, as it is trying to elicit an emotional response. You can use instead ‘may indicate that’.

*Response: We thank the reviewer for this comment and have revised the sentence to replace emotionally charged language with more neutral phrasing. The revised sentence now reads: “In Moshi, Tanzania, rates of alcohol use are 2.5 times higher than the national average, which may indicate that similar patterns of alcohol consumption extend to pregnant women.”*

**Comment:** “…, warranting further investigation15…” Is this your conclusion?

*Response: We thank the reviewer for this question. The original sentence was intended to reflect the notion that the higher rates of alcohol use in Moshi may indicate similar consumption patterns among pregnant women in the region. Our intention in using the phrase “warranting further investigation” was to highlight an identified gap in the literature regarding the limited knowledge of specific consumption patterns and drinking practices among pregnant women in Moshi. However, to reduce potential confusion, we have decided to remove the phrase “warranting further investigation,” given that we elaborate on this identified gap in the last two paragraphs of the introduction.*

**Comment:** 115 -120 Please add references.

*Response: We thank the reviewer for their careful examination of our references. We acknowledge this oversight in not including citations for these claims. We have conducted a comprehensive review of the manuscript and have now added the necessary citations to ensure the accuracy of all statements made.*

**Comment:** “…there is a limited understanding of alcohol use practices, the knowledge and attitudes related to ACDP, and the ways these impact cultural beliefs among both pregnant women and women of child-bearing age…” Some of these things have been researched before, even in the same region. Granted, this is a MSc. thesis, but there are many similarities. Please see the following: Mbuya, B.K., 2022. Factors contributing to drinking alcohol during pregnancy in rural Kilimanjaro Tanzania: a descriptive qualitative study.

*Response: We thank the reviewer for highlighting this very important study by Mbuya. Mbuya’s findings effectively illustrate how social and cultural contexts significantly influence a woman's decision to consume alcohol during pregnancy. We recognize the value of this research and will emphasize it in our revised manuscript, as it provides essential local insights. As the reviewer notes, our analysis shares similarities with Mbuya’s work in seeking to understand the knowledge, attitudes, community perspectives, and cultural beliefs impacting alcohol consumption during pregnancy in Northern Tanzania. It is important to recognize that Mbuya focuses exclusively on the perspectives from childbearing-aged and pregnant women. In their discussion, they call for a deeper exploration of the "culture" surrounding prenatal alcohol use in the region. In response to this call, our study works to expand on Mbuya’s research.*

*Specifically, we aim to broaden the scope of this investigation into the knowledge, attitudes, community perspectives, and cultural beliefs influencing prenatal alcohol use to include insights from the entire community, encompassing insights from various genders (e.g., men and women) and generations (e.g., younger and older). By reassessing the knowledge, attitudes, and community perspectives within this wider population, we will effectively build upon Mbuya’s findings.*

*Our revised manuscript now states, “Existing research on this topic in Northern Tanzania has assessed female experiences with drinking during pregnancy and the contextual factors contributing to these behaviors. We aim to expand on this information by exploring the specific consumption patterns and alcohol use practices among pregnant women, the knowledge and attitudes related to prenatal alcohol exposure across different genders and generations, and how community perceptions and cultural beliefs may influence these. Insights from Moshi community members —women and men, young and old— will inform the design of age- and gender-appropriate alcohol reduction interventions and bolster education efforts for reproductive-aged women, ultimately aiming to better support pregnant women and reduce alcohol-related pregnancy complications.” Overall, this broader analysis acknowledges that alcohol use is a generational issue deeply tied to cultural beliefs and social norms present not only among women of reproductive age but also within the wider community.*

**Comment:** “This information is needed…” you precede to make a few claims regarding the need for this information, however you have not provided any references.

*Response: We appreciate the reviewer in bringing this to our attention and acknowledge our oversight in not including citations for these claims. We have conducted a comprehensive review of the manuscript and have now added the necessary citations to ensure the accuracy of all statements made.*

**METHODS**

STUDY DESIGN OVERVIEW

**Comment:** “…focused on exploring unhealthy alcohol consumption at the KCMC ED and RHC.” Please change: ‘focused on the exploration of alcohol consumption among female patients attending KCMC ED and RHC’. In that way you are not implying people drinking in the hospital. Further, as all alcohol consumption is unhealthy and you are not differentiating between healthy and unhealthy alcohol consumption, it might be best to leave it out.

*Response: We thank the reviewer for this feedback. We understand your concern about the phrasing of this sentence. We have revised the sentence to clarify that the focus is on the exploration of alcohol consumption among patients attending the KCMC ED and RHC. Additionally, we have removed the reference to 'unhealthy' alcohol consumption to avoid any unintended implications. The sentence now reads: “This manuscript represents a sub-study originating from a sequential explanatory mixed-methods study conducted in Moshi, Tanzania —an urban town with over 200,000 residents— focused on exploring gender differences in alcohol use among patients attending the KCMC ED and RHC.”*

**Comment:** “The high concentration of injury patients…” do you mean injured patients?

*Response: We thank the reviewer for their question. The term ‘injury patients’ is used intentionally to refer to patients who have experienced injuries or physical trauma, and are receiving treatment at the KCMC ED. To make this more clear, we have revised the sentence which now reads, “The high concentration of patients presenting with a chief complaint of injury or physical trauma at KCMC’s ED is also a population known for exhibiting high-risk alcohol use behaviors.”*

**Comment:** “…provide ideal settings to study risky alcohol use behaviors…” Why? The previous claims in the sentence does not explain why this is the ideal setting. It merely explains that there are many coming in with injuries and that there are many women.

*Response: We thank the reviewer for highlighting this issue and providing the opportunity to clarify. The KCMC ED was selected as a clinical site due to the higher rates of alcohol consumption observed in its patient population compared to the general population. This setting enables us to capture instances of frequent and high-risk alcohol use among women, including pregnant women. Specifically, these patients often drink heavily, get injured, and then present to the KCMC ED with injuries or physical trauma due to alcohol-related incidents. This is the only clinical site in the region where this occurs, making this site ideal to target and preferentially enroll individuals with alcohol use disorders and better understand risky alcohol use behaviors, particularly among pregnant women.*

*The Reproductive Health Centre (RHC) was chosen for its exclusively female patient population, which includes a significant number of pregnant women, thereby offering a representative sample to study alcohol use among this demographic. Overall, these clinical settings were purposefully chosen to ensure a comprehensive representation of various drinking patterns (e.g., abstinence, moderate consumption, frequent and/or heavy consumption) among women, and pregnant women, in the region.*

*In response to this feedback, we have revised the sentence for improved clarity: “The high concentration of injury patients presenting with a chief complaint of injury or physical trauma at KCMC’s ED is also a population known for exhibiting high-risk alcohol use behaviors. Thus, the KCMC ED provides an ideal setting for studying women with frequent or heavy alcohol use. The RHC, which exclusively serves female patients, offers a representative sample for investigating varying drinking patterns among women. Together, these clinical settings allow for a comprehensive assessment of alcohol use patterns across different demographics of women, particularly pregnant women.”*

**Comment:** “Quantitative surveys and qualitative semi-structured in-depth interviews (IDIs) were conducted, with surveys used to guide purposeful qualitative data sampling.” Do you mean that the survey have influenced the qualitative interview guide that was used during the IDIs?

*Response: We thank the reviewer for the opportunity to elaborate. To clarify, the survey did not influence the qualitative interview guide used during the IDIs. The same interview guide was used for all 19 IDI participants. Instead, we employed a purposive sampling method to select participants for the in-depth interviews based on predetermined characteristics identified in the survey, such as gender, demographic backgrounds, and alcohol consumption behaviors. Participants were purposively selected to ensure a diverse and representative sample, balanced with regards to gender, diverse demographic backgrounds (e.g., age, religion, income, profession, education, and marital status), and varying degrees of personal alcohol consumption. We describe this purposive sampling method further in the Qualitative Data - Sampling section of the revised manuscript.*

*To reiterate this, we have revised the original sentence: “Out of all 655 participants who completed the survey, 19 were purposively selected —based on trends observed among initial survey responses— to take part in semi-structured in-depth interviews (IDIs).”*

**Comment:** “A total of 541 women were enrolled in this study, with 533 providing complete survey data.” With ‘enrolled’, do you mean ‘approached’, or ‘recruited’ or ‘fell within our criteria and were approached’?

*Response: We thank the reviewer for highlighting the need for clarification regarding the terms used. To better clarify, the 541 women referenced encompassed the individuals who sought care at the KCMC ED or RHC, met our inclusion criteria, were approached for participation, and then completed the quantitative survey. However, we utilized survey data from only the 533 women who reported both their pregnancy status and age, as these served as primary stratification variables for the quantitative analysis aimed at assessing consumption patterns, knowledge, and attitudes across different female demographics: pregnant women, women of reproductive age, and women not of reproductive age. Accordingly, this section now reads, “A total of 676 adult patients seeking care at either the KCMC ED or RHC met our inclusion criteria, were approached for participation using a systematic random sampling method and were enrolled. Among these patients, 541 women and 114 men completed the survey. For this manuscript, the final quantitative dataset comprised 533 women who reported both their pregnancy status and age.”*

**Comment:** “…totaling 10 female and 9 male IDI participants…”. Now you stated that this is in an attempt to provide a broader community perspective on the topic, but is this the only reason you interviewed nearly the same amount of men as women? Is the male point of view weighing this heavily in a research which focuses on a female health issue?

*Response: We appreciate the reviewer’s question. The nearly equal representation of men and women in the IDIs was deliberate. The first reason for including men relates to the notion that this manuscript represents a sub-study of a larger mixed-method study. In this larger study, the primary aim was to explore gender differences in alcohol use in the region, which required interviewing an equal number of men and women.*

*Secondly, for the purposes of this manuscript, including men aligns with our goal to explore the community’s knowledge, attitudes, and cultural beliefs about prenatal alcohol use from a gender-balanced perspective, rather than an exclusively female perspective.*

*The reviewer raises an important point about the weight of the male perspective in a study focusing on a female health issue. While women provided more detailed insights on prenatal alcohol use, as reflected by the higher number of female quotes in the qualitative results, male perspectives are equally important. For instance, men often play significant roles as family members, such as brothers and husbands, who influence the health behaviors and decision-making processes of women, including alcohol consumption during pregnancy. Additionally, including men helps to capture the broader societal and cultural contexts in which alcohol use behaviors occur and are perceived. Thus, while men had less to say specifically about this female health issue, their perspectives are still crucial for understanding the overarching societal and cultural themes present in the region which impact prenatal alcohol use, all of which are essential for designing effective interventions that consider the social dynamics influencing alcohol use during pregnancy.*

*We have clarified this in the revised sentence which now reads, “The larger study aimed to explore gender roles and alcohol use, necessitating an equal number of male and female IDIs. For this sub-study, men were also included to address overarching societal themes and provide a broader, gender-balanced community perspective on prenatal alcohol use. Thus, the IDI participants comprised ten females and nine males.”*

**Comment:** “A thorough overview of the methods has been previously published 17.” What do you mean? As these author appear to have done a similar, but definitely different research. Or have you used their research? They also have recruited different number of patients, according to that publication. Were you working side by side? Was there an overlap in your studies? May this have been of influence in the data obtained?

*Response: We thank the reviewer for these questions and the opportunity to clarify our statement. First, we acknowledge the ambiguity in our original phrasing. Our intention was to communicate that this manuscript represents a sub-study that stems from a larger mixed-methods study focused on understanding gender differences in alcohol consumption in Moshi, Tanzania.*

*For the purposes of this manuscript, we are focusing on a subset of the data. Specifically, we are utilizing (1) survey data from 533 women who have reported both their pregnancy status and age, which are our primary stratification variables for the quantitative analysis, and (2) qualitative data from 19 in-depth interviews (10 women and 9 men), focusing particularly on community knowledge, attitudes, and cultural beliefs surrounding alcohol use during pregnancy.*

*The previous publications and our current manuscript share the same initial participant pool but diverge in the specific topic and subset of data analyzed. Thus, there is no direct overlap in the research studies as our current manuscript is independently analyzing data related to pregnant women and their alcohol use, a specific focus not addressed in prior publications.*

*In light of all this, we have removed the original sentence, “A thorough overview of the methods has been previously published 17,” and instead wrote, “This manuscript represents a sub-study originating from a sequential explanatory mixed-methods study conducted in Moshi, Tanzania —an urban town with over 200,000 residents— focused on exploring gender differences in alcohol use among patients attending the KCMC ED and RHC.”.*

EXCLUSION CRITERIA

**Comment:** Why is this a separate heading within the overview, when you are discussing sampling later on?

*Response: We thank the reviewer for highlighting this and providing us with an opportunity to clarify. The section originally titled “Exclusion Criteria” in the “Study Design Overview” was meant to outline the eligibility criteria for survey participation for all adult patients—men and women—who attended the KCMC ED or RHC. In the revised manuscript, we have removed the section heading “Exclusion Criteria” along with its related content; instead, we integrated this information into the “Quantitative Data - Procedures” section to clearly outline the pre-selection criteria used to determine which patients were approached for study participation.*

*To further clarify, the sampling discussed later in the “Qualitative Data - Sampling” section refers to the purposive qualitative sampling strategy we employed to select participants for in-depth interviews, which was based on their survey responses. We touch upon this in upcoming responses.*

**Comment:** “…not prisoners…” Why not? They may have had even more experience with alcohol use. Was there a large female prisoner contingency, which warrant this exclusion criteria?

*Response: We thank the reviewer for this comment. We agree with the reviewer that female prisoners might have relevant experiences with alcohol use. However, the decision to exclude prisoners from the study was largely based on ethical considerations and logistical constraints. Specifically, our aim was to ensure that all participants could provide strong, voluntary consent. Given their position of greater societal vulnerability, prisoners may face challenges in providing fully autonomous consent. Additionally, there are numerous laws and regulations that govern research involving incarcerated individuals in Tanzania, which would have posed significant logistical challenges. Nonetheless, we have taken note of your comment and will consider it for future research directions.*

**Comment:** “…fluent in Kiswahili,…” Kiswahili or Swahili is the official language beside English, however many other languages are spoken in Tanzania. Were there many that were rejected to participate as a result of this? Did this perhaps alter the sample population, as the other language speaking communities in the region may have given a different point of view within the same area?

*Response: We thank the reviewer for their thoughtful questions. This inclusion criterion did not alter our sample population, and no participants were rejected to participate as a result of this. To be clear, all patients approached for study participation were fluent in Kiswahili. While English and other languages are also spoken in Moshi, Tanzania, they are not as universally understood in this area as Kiswahili.*

**Comment:** “Literature defines the reproductive lifespan of women as 45 years and below45.” I assume this is the wrong reference.

*Response: We have revised the reference error pointed out by our reviewer. The error was due to a mistake in the use of the reference manager for this manuscript. We have since added the correct references for this statement and throughout the manuscript.*

QUANTITATIVE DATA

PROCEDURE

**Comment:** “Using intake triage registries from the ED and the RHC, a systematic random sampling approach was used to approach every third patient seeking care…”. I think you probably would have done a pre-selection of the registry, to only include those within the criteria of your research?

*Response: We thank the reviewer for this important question. You are correct that all patients over the age of 18 and who were not prisoners were initially pre-screened for using intake triage registries from the KCMC ED and RHC. Only participants meeting both of these criteria were then approached using a systematic random sampling method (i.e., every third patient seeking care who fell within these initial inclusion criteria was approached). Further inclusion criteria (e.g., fluency in Kiswahili and the ability to provide informed consent) were assessed upon approaching the patient. We have revised this section of the manuscript, which now states, “All patients who were (1) over the age of 18 and (2) not prisoners were initially screened for using intake triage registries from the KCMC ED and RHC. Using a systematic random sampling method, every third patient meeting these two criteria was approached. The remaining inclusion criteria — particularly fluency in Kiswahili and the ability to provide informed consent — were assessed upon approaching the patient.”*

**Comment:** “Gender-matched Tanzanian research assistants…” this is phrase that I have not seen used in academic research before with the meaning that you are intending. Often when gender-matched is used it is referring to data in a cohort. For clarity, as within this chapter, you are solely getting “females” to answer a survey, would continuing the term ‘female’ be better?

*Response: We thank the reviewer for sharing their knowledge and suggestions. We agree and recognize that the term "gender-matched" may not accurately convey our intent. In light of your comment, we have revised the sentence to read: “Female research assistants (RAs) fluent in Kiswahili provided an overview of the study to female patients in a private clinic setting, while male RAs did the same for male patients.”*

INSTRUMENTS

**Comment:** “The Tanzanian research team translated, reviewed, revised, and pilot-tested all survey questions on sociodemographic data and self-reported alcohol use prior to data collection.” There are many different steps in translating research tools. How were the translated tools validated?

*Response: We appreciate the reviewer’s important question regarding the translation and validation process. The basic demographic tools, which included survey questions on sociodemographic data and self-reported alcohol use, were translated from English to Swahili by research assistants fluent in both English and Swahili; these research assistants also had prior experience in translation work. These translations were pilot-tested, in order to ensure that they (1) were properly phrased with correct grammatical syntax, and (2) maintained the same meaning in Tanzania as originally written in English. Any errors identified during pilot-testing were addressed before participant enrollment began. Overall, these basic tools consisted of demographic and self-reported alcohol use questions that were straightforward enough that extensive translation work was not required. To better communicate this in the updated manuscript, we have instead written, “The Tanzanian research team, fluent in English and Kiswahili and experienced in language translation, translated all basic survey questions on sociodemographic data and self-reported alcohol use from English to Kiswahili. Prior to data collection, these translations were pilot-tested and revised to ensure grammatical accuracy and the preservation of the original meanings within the basic survey tools.”*

*For the more complex tools used in this study, such as the AUDIT, DrInC, and PHQ-9 surveys, they underwent thorough clinical and psychometric validation in the region, as cited in the manuscript.*

QUALITATIVE DATA

SAMPLING

**Comment:** “…since data saturation is typically reached around 20 interviews…” I am unable to find and read the books that you have referenced here. However, it is unlikely that they would give a number.

*Response: We appreciate this comment from the reviewer. The qualitative research community generally supports the understanding that a range of 9 to 17 in-depth interviews (IDIs) is often sufficient to reach saturation when the aim of the topic is narrow in scope, as stated by Hennick and Kaiser. Given this, the research team maintained high certainty that saturation would be reached within 20 IDIs. Additionally, a sample size of 20 IDIs was chosen as it aligned with the study timeline and goals. In light of these points, we have alternatively written, “Specifically, we aimed to interview ten males and ten females, based on qualitative research recommendations suggesting that data saturation is typically reached within a range of 9 to 17 IDIs for narrowly focused topics.” We hope this edit resolves any misunderstanding regarding our qualitative sample size estimation.*

**Comment:** “However, saturation was reached with 19 IDIs, comprising nine males and ten females” You are missing a period at the end.

*Response: We acknowledge this grammatical error, along with others throughout our manuscript. We have corrected these mistakes and conducted a comprehensive review of the entire manuscript for English and grammar. All identified errors have been addressed.*

**Comment:** You described that there were multiple demographical difference between the interviewees, these being: age, marital status, education level, occupation, tribe, religion, sex, pregnancy status. Of these 8 characteristics, do you say that you have obtained saturation of data until a point where no new data would appear?

*Response: We appreciate the reviewer’s question and would like to clarify. The eight characteristics mentioned by the reviewer were used to ensure a representative and diverse sample via the purposive sampling strategy previously mentioned, not to achieve data saturation on each individual characteristic.*

*As previously mentioned, predetermined characteristics identified in the survey, such as demographic backgrounds (e.g., age, religion, income, profession, education, and marital status), gender, and perspectives on and experiences with alcohol were considered when purposively selecting individuals for IDI participation, to ensure a diverse and representative sample. The consideration of these characteristics was not used to determine whether data saturation was reached.*

*Instead, data saturation was defined as the point at which no new information or themes regarding community perceptions on prenatal alcohol use emerged after three consecutive interviews. This saturation point was reached after conducting 19 IDIs, consisting of nine males and ten females. That is to say, it was thematic saturation to which we were referring to in the original sentence; saturation was not based on demographic variables. We have revised the paragraph to clearly define how we identified whether data saturation was obtained.*

PROCEDURES

**Comment:** “Upon completion of the quantitative survey, research assistants identified suitable candidates for IDIs using their professional judgment…” You stated before that only women took part of the quantitative survey, so how did they identify the men that took part in the qualitative IDIs?

*Response: We thank the reviewer for the opportunity to clarify and recognize how this may have caused confusion. As previously mentioned, this manuscript originates from a larger, sequential explanatory mixed-methods study, in which both men and women participated in the quantitative survey. For the purposes of this manuscript, our quantitative analysis focused only on data from 533 women who provided their pregnancy status and age to explore alcohol use practices among pregnant women, women of reproductive age, and women not of reproductive age. However, men also completed the quantitative survey, and these data were used to identify male participants for the qualitative IDIs. A purposive sampling method was employed to ensure a diverse and representative sample, balanced with regards to gender, diverse demographic backgrounds (e.g., age, religion, income, profession, education, and marital status), and varying degrees of personal alcohol consumption. We have made significant revisions to the methods section of this manuscript to more clearly communicate our procedures.*

**Comment:** “Using their professional judgement” is a sure fire way to cherry pick data.

*Response: We thank the reviewer for this comment and acknowledge the concern regarding the use of the term “professional judgment.” We have since revised this paragraph to better clarify the steps taken to purposively select IDI participants, specifically through the sampling technique described in the comment above. We also want to reiterate that steps were taken to minimize sampling bias in participant selection for the semi-structured in-depth interviews (IDIs). The revised manuscript states, “Upon completion of the quantitative survey, research assistants invited suitable candidates for IDIs based on predetermined characteristics identified in the survey. Those invited were purposively selected to represent diverse sociodemographic backgrounds, perspectives, and experiences related to alcohol… To minimize unintentional sampling bias, the study lead reviewed interviewee characteristics monthly, with necessary adjustments in participant selection implemented immediately.”*

**Comment:** “…with monthly reviews of sociodemographic and alcohol-use-related data conducted to ensure diverse representation of participants.” So did they select based on this data? What did they select for? People who were most represented in the list? What were the points of data in this list?

*Response: We thank the reviewer for this important question. To clarify, the purposive selection of IDI participants was based on (1) sociodemographic statuses (e.g., age, religion, income, profession, and marital status), (2) alcohol consumption levels, and (3) perspectives and experiences related to alcohol, all of which were recorded during the survey collection. For instance, regarding perspectives on alcohol, we considered individuals with a variety of experiences: those who reported neutral or positive past experiences, individuals who previously consumed alcohol but are now abstinent, those with close friends or family members who drink heavily, and those who have encountered significant negative consequences from alcohol use. Overall, our aim was to select individuals to create the most diverse sample possible across these variables, ensuring that the range of community perspectives on prenatal alcohol use was well-represented in the IDIs.*

**Comment:** “Interviewers were fluent in Kiswahili, gender-matched,…” see previous comments on both topics.

*Response: We thank the reviewer for bringing this to our attention. The sentence now reads, “Interviewers were of the same gender as the interviewee and fluent in Kiswahili.”*

INSTRUMENT

**Comment:** “…with probes built in for additional questioning when the subject matter was ambiguous, vague, interesting, or inconsistent.” When you say subject matter, do you mean the interviewee response?

*Response: We appreciate the reviewer’s question and the opportunity to clarify. In this context, we are indeed referring to the interviewee's responses, and we have revised the sentence accordingly: “... with probes built in for additional questioning when the interviewees’ responses were ambiguous, vague, interesting, or inconsistent.”*

**Comment:** “Cultural appropriateness and relevancy were ensured during pilot testing.” How? Is this not an exploration of the cultural perceptions in regards to the subject?

*Response: We appreciate the reviewer’s question and recognize that the original sentence was confusing in its phrasing. Our statement regarding "cultural appropriateness and relevancy" does not refer to our exploration of cultural perceptions related to prenatal alcohol use. Rather, it pertains to the careful review of phrasing and syntax during the translation process to ensure that the original meaning of the open-ended questions was retained, given the differing contexts (e.g., English as used in the United States, and Kiswahili as used in Tanzania). This translation process ensured that the translated open-ended questions were relevant, culturally appropriate in their phrasing, and understandable within the Tanzanian context. We have revised this sentence to better communicate this idea: “The translated, open-ended questions were reviewed and revised for proper phrasing and syntax. These translations were then pilot tested to ensure they were relevant, culturally appropriate in their phrasing, and understandable within the Tanzanian context.”*

ANALYSIS

**Comment:** “Qualitative data was analyzed using an applied, thematic, grounded theory approach” These are different ways of analysing. If you used GT, which version?

*Response: We thank the reviewer for these thoughtful questions. We recognize that multiple qualitative analysis methods were originally listed. To better clarify, we used the classical version of the grounded theory approach. This method is ideal for emergent topics where no initial literature exists within the problem area and there is little understanding of the social processes at work, as was the case at the start of the broader study. With regards to the conduct of research, a draft of our codebook was developed both deductively and inductively after the first four interviews. The interview guide provided broad categorical domains deductively, while subsequent analysis of the interview transcripts allowed us to inductively generate specific themes from the data without preconceived theoretical hypotheses. This "laissez-faire" approach characteristic of classic grounded theory allowed the themes to emerge organically from the data with minimal researcher intervention. The codebook remained dynamic and non-linear and was updated as new themes emerged throughout data collection.*

*This section now reads, “Qualitative data was analyzed using a classical grounded theory approach. Due to the limited research on pregnant female drinking behavior in Moshi, this approach was particularly suitable for capturing and exploring themes arising organically. That is, the classical grounded theory methodology facilitated the generation of themes directly from the transcripts without relying on preconceived theoretic frameworks, ensuring that the findings were rooted in the participants’ experiences and perspectives.” To support this statement, we have added a reference highlighting classical grounded theory, which guided our research conduct and evaluation: Hunter A, Murphy K, Grealish A, Casey D, Keady J. Navigating the grounded theory terrain. Part 2. Nurse Res. 2011;19(1):6-11. doi: 10.7748/nr2011.10.19.1.6.c8765. PMID: 22128581.*

**Comment:** “Considering research on pregnant female drinking behavior is limited in Moshi, the grounded theory approach was well-suited to capture emerging themes.” Please explain what you mean by this.

*Response: We thank the reviewer for allowing us to elaborate. At the start of our broader study, there was limited research attention on female drinking behaviors in Moshi, Tanzania. Specifically, alcohol use practices during pregnancy, as well as knowledge and community perspectives on prenatal alcohol use in this region, were unstudied topics with no existing publications addressing them. Thus, a classical grounded theory approach was deemed appropriate to study prenatal alcohol use from the perspectives of both pregnant women and the wider community, considering this methodology is well-suited for exploratory research. The classical grounded theory approach allowed us to capture new themes arising organically and directly from the data (e.g., transcripts) provided by community members themselves. Findings were rooted in the participants' experiences and perspectives. We have revised the manuscript to clarify this and have addressed it in the previous response as well.*

**RESULTS**

QUANTITATIVE

**Comment:** “Notable differences in marital status were also observed between pregnant women and non-pregnant women.” If it is notable, please share what it is.

*Response: We thank the reviewer for their comment. The notable differences in marital status between pregnant and non-pregnant women are described in the two sentences following this statement. To avoid confusion, we have decided to remove the initial sentence and directly describe the differences in marital status.*

**Comment:** “…those who were more pregnant were more likely to be living with a partner…” More pregnant?

*Response: We acknowledge this grammatical oversight and have corrected it accordingly.*

**Comment:** “…than older, non-childbearing women.” So you are pointing out that women aged 17-45 have different characteristics than women aged 45-95. Were you expecting those in the ages above 70 to still be employed?

*Response: We appreciate the reviewer's comment. While we recognize that the employment rates for women above 70 are typically lower, our analysis focuses on the overall trends observed in the broader age group of women not of childbearing age, who average around 60 years old. In this comparison, we aim to illustrate how younger women, especially those who are pregnant, are increasingly achieving higher educational and income levels compared to older, non-childbearing women. This highlights the shifting socioeconomic landscape (e.g., recent trends in girls' education) and its implications for health outcomes. Particularly, these improvements in women’s education in Moshi may also help to spread community-wide health messaging and pre-pregnancy education related to prenatal alcohol use. Thus, we believe this socioeconomic context is essential for understanding the potential for breaking cycles of generational harm associated with alcohol use during pregnancy, a point we bring up in our discussion.*

**Comment:** Line 262-264 why are you comparing drinking frequencies between young pregnant women to those not of childbearing age? That is comparing apples with oranges.

*Response: We appreciate the reviewer’s comment regarding the comparison of drinking frequencies between young pregnant women and those not of childbearing age. While this may seem like comparing "apples to oranges," our goal was to highlight differences in personal drinking patterns, as well as many other comparisons, such as knowledge and attitudes toward prenatal alcohol use, across these female groups.*

*The findings from our comparison of these three groups—pregnant women, non-pregnant women of childbearing age, and non-pregnant women not of childbearing age—demonstrate how different female demographics experience and perceive alcohol use. Understanding these differences is crucial for informing the development of tailored community messaging, educational initiatives, and interventions aimed at reducing prenatal alcohol exposure (as discussed in our discussion). Such efforts should target not only pregnant women but also young women before they become pregnant, as well as older women who act as cultural arbiters and educators for the younger generation.*

*Thus, we believe this comparison enriches our findings and contributes to the contextual framework for future implementation research addressing prenatal alcohol exposure across various generations of women (e.g., present and future). By emphasizing the unique rates of use and other comparisons among these three groups, this analysis offers information relevant for breaking generational cycles of harm associated with alcohol consumption during pregnancy.*

**Comment:** There are other points in this part where you compare groups who differ on more than one parameter.

*Response: We appreciate the reviewer for highlighting the complexity of our comparisons among different groups. We acknowledge that the three female groups we compare differ on multiple parameters, including age and pregnancy status. Our aim in these comparisons is to highlight the multifaceted nature of alcohol use among women, emphasizing how each group may indirectly influence prenatal alcohol exposure. We believe that examining these groups collectively allows us to identify broader trends and patterns critical for understanding prenatal alcohol consumption in the region, especially considering that prenatal alcohol use represents a generational harm, which we bring up in the discussion section of this manuscript.*

**Comment:** Line 283 till 285, please move to above the table.

*Response: We thank the reviewer for bringing this formatting mistake to our attention and recognize this oversight. This has been corrected.*

**Additional comment from the authors:** In response to medRxiv's request, we removed participant ages from the qualitative results section (e.g., quotes) to protect identifying information. We want to emphasize that the IDI numbers (IDI #1 - IDI #19) associated with these quotes were known only to the research personnel and, therefore, were not removed; this was clarified in the methods section.
